# Effect of Thickness of HA-Coating on Microporous Silk Scaffolds Using Alternate Soaking Technology

**DOI:** 10.1155/2014/637821

**Published:** 2014-06-29

**Authors:** Hongguo Li, Rui Zhu, Liguo Sun, Yingsen Xue, Zhangying Hao, Zhenghong Xie, Xiangli Fan, Hongbin Fan

**Affiliations:** ^1^Institute of Orthopedic Surgery, Xijing Hospital, The Fourth Military Medical University, Xi'an 710032, China; ^2^Department of Orthopaedics, 513 Hospital of PLA, Lanzhou 732750, China; ^3^College of Science, Air Force Engineering University, Xi'an 710032, China; ^4^Department of Military Medical Training, Comprehensive Training Base of Lanzhou, Hutubi 831200, China

## Abstract

Hydroxyapatite (HA) can be coated on various materials surface and has the function of osteogenicity. Microporous silk scaffold has excellent biocompatibility. In this study, alternate soaking technology was used to coat HA on microporous silk scaffolds. However, the cell proliferation was found to decrease with the increasing thickness (cycles of soaking) of HA-coating. This study aims to determine the best thickness (cycles of soaking) of HA-coating on microporous silk scaffolds. The SEM observation showed that group with one cycle of alternate soaking (1C-HA) has the most optimal porosity like non-HA-modified microporous silk scaffolds. The proliferation of osteoblasts has no significant difference between noncoated HA (N-HA) and 1C-HA groups, which are both significantly higher than those in two cycles of soaking (2C-HA) and three cycles of soaking (3C-HA) groups. The transcription levels of specific genes (*runx2* and *osteonectin*) in osteoblasts of 1C-HA group were significantly higher than those of N-HA group. Moreover, the levels showed no significant difference among 1C-HA, 2C-HA, and 3C-HA groups. In conclusion, microporous silk scaffold with 1 cycle of HA-coating can combine the biocompatibility of silk and osteogenicity of HA.

## 1. Introduction 

Hydroxyapatite (HA, Ca10(PO4)6(OH)2) is one of the most disquisitive biomaterial. Their composition and crystal structure are similar to inorganic phase of bone. So HA has exceptional biocompatibility [1]. HA also possess excellent osteoinductivity and osteoconductivity [2–4]. Bone (65%) and tooth structure (97%) contain apatite mineral commonly known as HA [5]. HA-coating technology was widely used in the fabrication of tissue-engineered scaffolds. Alternate soaking technology is one of the most common methods to coat HA on materials. This method is especially suitable for modification of silk scaffolds [6].

Silk proteins are biosynthesized by epithelial cells and secreted into the specialized glands of silk worms. The silk proteins were stored in the silk glands until spun into fibers [7]. Bombyx mori silk is the most popular silkworm silk. It consists of two fibroin threads adhered together with sericin gum. A single thread is about 10–25 *μ*m in diameter [7, 8]. Silk fibroin (SF) fibers from B. mori have outstanding mechanical properties and little catalytic and molecular recognition. So they have been used as biomedical suture material for a long time [7]. SF has a lot of unique properties including excellent biocompatibility, favorable oxygen permeability, and outstanding biodegradability, and the degradation product can be readily absorbed with minimal inflammatory reactions [9, 10]. SF and microporous silk scaffolds have been used in various biomedical research fields including osteoblast, fibroblast, or bone marrow stem cell supported matrix and ligament tissue engineering [6, 11]. Moreover, it is very promising that biomimetic self-assembly process can be applied in tissue replacement materials of SF [12–14]. Silk modified with HA-coating can combine the biocompatibility of silk and osteogenicity of HA. In our pilot study, too thick HA-coating was found to have negative effects on cell proliferation and differentiation. This study was designed to find out an appropriate thickness of HA-coating which was beneficial for both cell proliferation and osteogenicity.

## 2. Material and Method

### 2.1. Fabrication of Microporous Silk Scaffold

The knitted silk scaffolds (2 × 4 cm in dimension) were fabricated using raw silk fibers (Suzhou Silk Factory, China) with knitting machine (Silver-reed SK270, Suzhou, China). The knitted scaffolds were degummed in a solution of 0.25% (w/v) NaCO_3_ between 98 and 100°C for 90 min to remove the sericin coated on the silk fibroin. The solution should be refreshed after 45 min. The degummed silk scaffolds were then rinsed with distilled water for 1 h to remove any residual degumming solution and then air-dried.

Degummed silk fibers were used to prepare silk fibroin solution. They were dissolved in 9.3 M LiBr solution with a ratio of 1 : 4 (1 g/4 mL) at 60°C for 4 h with continuous stirring [15]. The resulting silk solution was dialyzed against distilled water for a period of 48 h with SnakeSkin (Thermo Scientific Co., 3500 MWCO, USA). The concentration (w/v) of the dialyzed solution was determined by measuring the weight of silk fibroin obtained by freeze-drying.

The degummed silk scaffold was immersed in the 2% w/v silk solution and freeze-dried for 24 h to allow formation of microporous silk sponges. The sponge-coated hybrid scaffolds were treated with 90/10 (v/v) methanol/water solution for 10 min to induce an amorphous to silk II conformational change in the microporous sponges to prevent resolubilization in cell culture medium. Finally, the hybrid scaffolds were dried overnight in a fume hood. Thereafter, the scaffold was cut into round disc (diameter: 15 mm) to fit for the well size of 24-well culture plate.

### 2.2. Hydroxyapatite- (HA-) Coating on Hybrid Silk Scaffold

**Figure 1:**
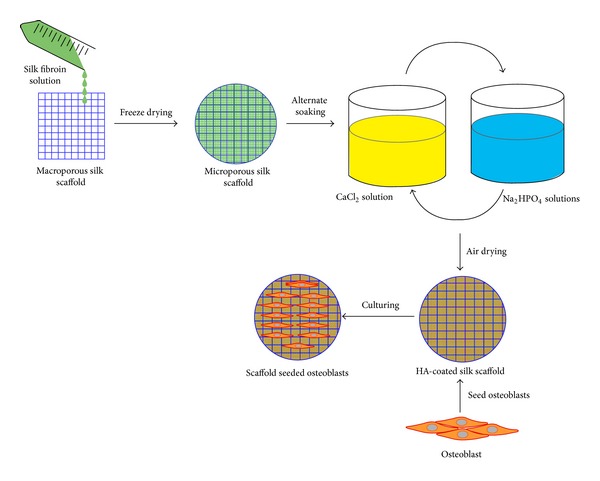
Schematic outline of scaffold fabrication procedure and experimental process.

The hybrid silk scaffold was coated with hydroxyapatite using an alternate soaking technology [6]. Briefly, the silk scaffold was immersed in 200 mM calcium chloride (CaCl_2_) solution in a Petri dish placed at 37°C for 1 h. The scaffold was then blotted on a filter paper to remove excess moisture and then immersed in a 120 mM disodium hydrogen phosphate (Na_2_HPO_4_) solution under the same conditions for 1 h. The scaffolds were divided into three groups, in which the soaking process was conducted for one, two, and three cycles, respectively. After soaking process, the HA-coated silk scaffolds were washed in distilled water and air-dried at room temperature for 24 h (Figure 1). There were four groups assessed in present study and they were noncoating HA group (N-HA), one-cycle HA-coating group (1C-HA), two-cycle HA-coating group (2C-HA), and three-cycle HA-coating group (3C-HA).

### 2.3. Cell Isolation and Expansion

Osteoblasts were isolated via sequential collagenase digestion of neonatal rabbit calvaria according to established protocol [16]. They were cultured at 37°C in a humidified atmosphere of 5% CO_2_, in 50 cm^2^ flasks containing 5 mL Dulbecco's modified Eagle medium (Gibco) and 10% fetal bovine serum (Gibco). The medium was changed every third day. For subculture the cell monolayer was incubated with trypsin-EDTA solution (0.25% trypsin, 1 mM EDTA; Gibco) for 10 min at 37°C to detach the cells. Then, the cells were washed twice by centrifugation and suspended in complete medium for seeding and growing in new culture flasks. Osteoblasts at population numbers 2 (passage 2) were used in experiments.

### 2.4. Cell Proliferation and Collagen Production on Scaffolds

Cell proliferation of each group was studied by Alamar Blue colorimetric assay (Sigma, U.S.A.) and DNA content assay. The osteoblasts were seeded on scaffolds at a cell density of 2.0 × 10^5^ cells/cm^2^. Cell viability and proliferation on scaffolds were studied at 2, 7, 14, and 21 days after seeding using the Alamar Blue colorimetric assay. The scaffolds (*n* = 5) of each group were incubated in 1 mL of HG-DMEM supplemented with 10% FBS and 10% (v/v) Alamar Blue dye for 3 h. The absorbance of the culture media at 570/630 nm was measured in triplicates using a 96-well plate microplate reader. Using culture medium supplemented with 10% Alamar Blue dye as a reagent blank, the percentage of Alamar Blue reduction was calculated according to the formula provided by vendor. The DNA amount was quantified with Hoechst Dye 33258. Briefly, the cells were harvested from scaffolds (*n* = 5) of each group by incubating with 0.05% trypsin and lysed in cell lysis buffer after 2 weeks postculturing. Cell lysates were diluted 10 times and incubated with equal volume of 0.1 mg/mL Hoechest 33258 (Invitrogen, US) solution for 10 min at room temperature in 96-well black plates. Fluorescence was determined using a FLUOstar Optima fluorescent plate reader (BMG Labtech, Offenburg, Germany). The relative fluorescence unit value obtained from samples was extrapolated against a DNA standard curve to determine the DNA amount. The collagen production on scaffolds was quantified using Sircol collagen dye binding assay Kit (Biocolor Ltd., Newtown, Ireland). Briefly, scaffolds were digested with 500 *μ*L of pepsin solution (0.25 mg/mL) at 2 weeks postseeding. The suspension was shaken at room temperature for 2 h. Then 1 mL of dye reagent was added to 300 *μ*L of digested solution and mixed for 30 min at room temperature. The pellet of dyed collagen was precipitated by centrifugation at >10 000 g for 10 min and then dissolved by 1 mL of releasing reagent. The absorbance of redissolved dye was measured in 96-well plates at absorbance wavelength of 540 nm. Then collagen amount was extrapolated from standard curve. The collagen in each sample was presented as an amount normalized to the DNA content.

### 2.5. Cell Viability Study by Fluorescence Staining

FDA/PI double stain can distinguish live and dead cells. The fluorescein diacetate (FDA) solution (10 g/L) was prepared by dissolving FDA (Sigma) in acetone with the aid of a vortex mixer. The FDA solution was stored at −20°C until use. FDA solution (11 *μ*L) was added into 1 well of 24-well culture plate containing cells/scaffolds and 1 mL of culture medium. Thus, the final concentration of FDA was 100 mg/L. The PI solution (1 g/L) was prepared by dissolving propidium iodide (PI) (Sigma) into Milli-Q water and stored at 4°C until use. The PI solution (22 *μ*L) was added into 1 well of 24-well culture plate as mentioned above. Thus, the final concentration of PI was 20 mg/L. It was stained for 5 minutes under the condition of dark. The culture medium was discarded and the scaffolds were washed twice using PBS solution gently. The living and dead cells were observed with confocal laser scanning (Olympus fluo viem FV1000).

### 2.6. Morphology of Cells/Scaffolds

The cell morphology of osteoblasts was observed by HITACHI S-4800 (Japan) scanning electron microscopy (SEM). After three weeks of culture, the cells/scaffolds were washed with PBS and fixed in 2.5% (v/v) formaldehyde for 24 h followed by dehydration using graded ethanol. Then the dried cells/scaffolds were coated with gold using a Sputter Coater for 60 s at a current of 30 mA. Their morphology was observed with SEM at a voltage of 10 kV.

### 2.7. Quantitative RT-PCR Analysis of the Gene Expression

After three weeks of culture, gene expression analysis was performed to investigate the effects of HA-coating on the gene level of osteoblasts. Total RNA was extracted from each scaffold (*n* = 3) using Qiagen RNeasy Kit (Qiagen, Valencia, CA, USA). Thereafter, the purity and concentration of RNA were determined by UV-spectrophotometry (S2100 Diode Array Spectrophotometer, Biochrom, Cambridge, USA). cDNA synthesis was carried out using 80 ng of total RNA and reverse transcriptase (iScript, Bio-Rad Laboratories, CA, USA) with oligo (dT) primers. Quantitative reverse transcriptase-mediated-PCR (Q-RT-PCR) was performed using SYBR-Green chemistry (iQ SYBR Green Supermix, Bio-Rad) in an iCycler iQ detection system (Bio-Rad), with glyceraldehyde three-phosphate dehydrogenase (*GAPDH*) as reference genes. Gene expression of different gene markers was analyzed. Osteoblasts related gene markers involved Runt-related transcription factor 2 (*runx2*) and* osteonectin*  (ON). The primer sequences of selected genes for real-time PCR were obtained from the published literature [13] (Table 1). The amplification was performed in triplicate and data were analyzed for relative expression using the ΔΔCT method. The results were normalized to* GAPDH* gene expression levels.

### 2.8. Statistical Analysis

All sample values were expressed as the mean ± standard deviation (SD) and the data were analyzed using SPSS 10.0 software. Statistically significant values were defined as *P* < 0.05 based on one-way analysis of variance (ANOVA).

## 3. Result

### 3.1. Cell Proliferation and Collagen Production

**Figure 2:**
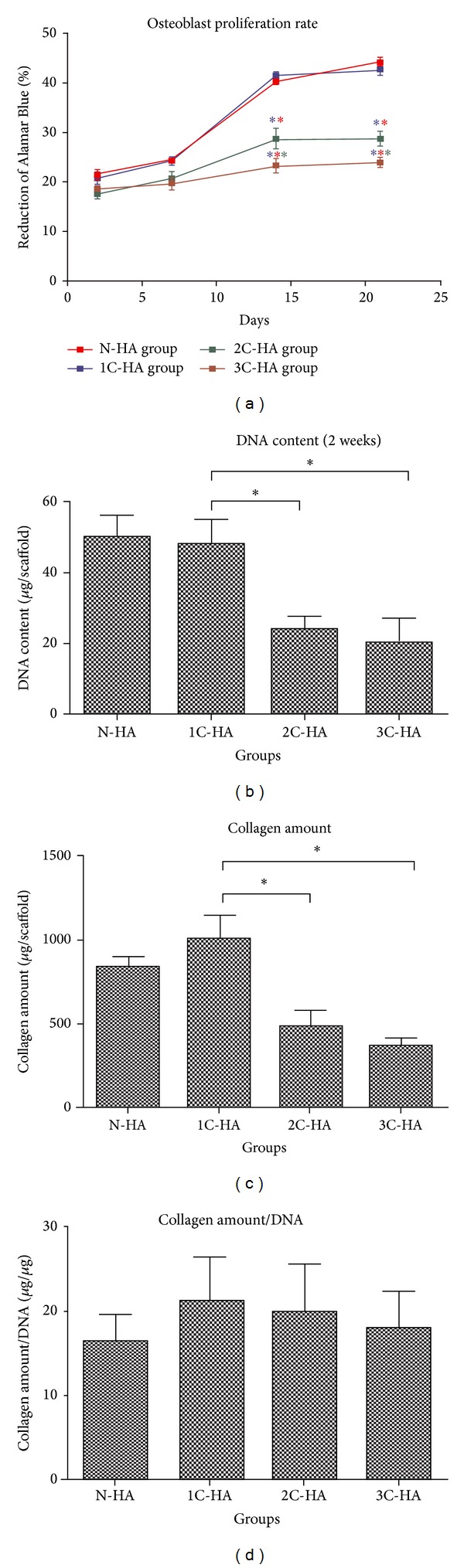
(a) Cell proliferation rate of N-HA, 1C-HA, 2C-HA, and 3C-HA groups during a 3-week culture period. (b) DNA content of osteoblasts on scaffolds of each group after 2 weeks postculturing. (c) Quantification of collagen production on scaffolds of each group after 2 weeks postculturing. (d) Quantification of collagen normalized by DNA amount on scaffolds of each group after 2 weeks postculturing. (*P < 0.05, ANOVA).

The proliferation and metabolism of osteoblasts in the four experimental groups were compared using Alamar Blue assay. In the first two weeks, the value of Alamar Blue reduction increased rapidly. After 2 weeks, it approximately doubled in both N-HA and 1C-HA groups with 40.2 ± 2.4% and 41.5 ± 1.9%, respectively. In contrast, the values increased more slowly in 2C-HA and 3C-HA groups with 28.7 ± 2.7% and 23.3 ± 2.1%, respectively. Then, the values increased continuously but at a slower rate. There was no significant difference in N-HA and 1C-HA group. But the values of N-HA and 1C-HA groups were both significantly higher than those of 2C-HA and 3C-HA groups after 2 weeks. Furthermore, the value of 2C-HA group was significantly higher than that of the 3C-HA group after 2 weeks (Figure 2(a)).

The DNA contents of osteoblasts on scaffolds of N-HA and 1C-HA groups were significantly higher than those of 2C-HA and 3C-HA groups (*P* < 0.05) with 50.2 ± 6.2 *μ*g and 48.2 ± 7.2 *μ*g after 2 weeks, respectively. The contents were 24.2 ± 3.6 *μ*g and 20.4 ± 7.0 *μ*g in 2C-HA and 3C-HA groups per scaffold after 2 weeks, respectively (Figure 2(b)). The amount of deposited collagen on scaffold was determined as an indication of extracellular matrix (ECM) formation. After 2 weeks of culture, collagen amounts on scaffolds of N-HA and 1C-HA groups were significantly higher (*P* < 0.05) than those of 2C-HA and 3C-HA groups, with 833.3 ± 66.6 *μ*g and 1008.3 ± 138.4 *μ*g, respectively. The collagen amount was 480.0 ± 48.4 *μ*g in 2C-HA group and 363.3 ± 49.3 *μ*g in 3C-HA group (Figure 2(c)). After being normalized against DNA content, the collagen amounts were 16.3 ± 3.2 *μ*g/*μ*g, 21.3 ± 5.2 *μ*g/*μ*g, 20.1 ± 5.6 *μ*g/*μ*g, and 18 ± 4.4 *μ*g/*μ*g in N-HA, 1C-HA, 2C-HA, and 3C-HA groups, respectively. The value of each group showed no significant difference (Figure 2(d)).

### 3.2. Morphology Observation of the Scaffolds

**Figure 3:**
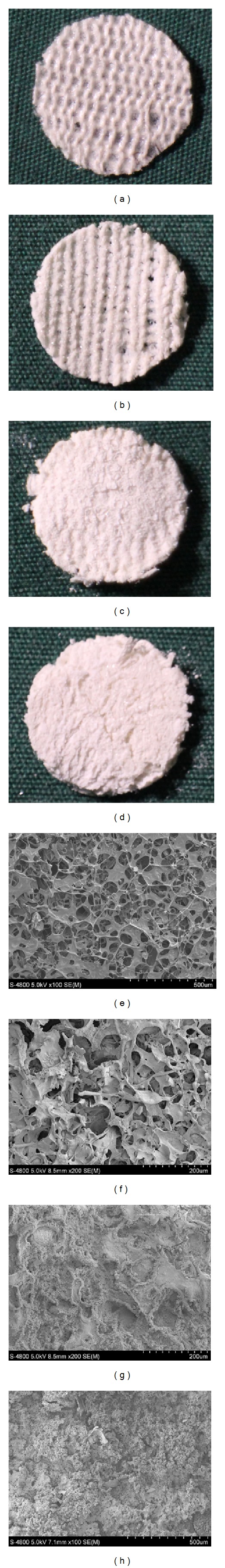
(a)–(d) Macroscopic view of scaffolds in N-HA, 1C-HA, 2C-HA, and 3C-HA groups. (e)–(h) SEM observation of the surface of scaffolds in N-HA, 1C-HA, 2C-HA, and 3C-HA groups.

Gross observation (Figures 3(a)–3(d)) showed that the thickness of HA-coating increased with the soaking cycles. The macroscopic view of 1C-HA group (Figure 3(b)) was similar to that of N-HA (Figure 3(a)) group. The thickness of coating in 2C-HA (Figure 3(c)) and 3C-HA (Figure 3(d)) groups significantly increased compared with those of 1C-HA and N-HA groups. SEM images (Figures 3(e)–3(h)) demonstrated an interconnected microporous silk sponge formed and spread over the surface of the knitted scaffold in N-HA (Figure 3(e)) and 1C-HA (Figure 3(f)) groups. The pore sizes of silk sponge ranged from 60 to 200 *μ*m. The average pore size of the hybrid silk scaffold was 101.5 ± 32.1 *μ*m. Nanoscale HA particles were successfully deposited and distributed on the surface of silk sponge as observed by SEM. The micropores had been filled up with HA in 2C-HA (Figure 3(g)) and 3C-HA (Figure 3(h)) groups. Especially in 3C-HA group, the surface of scaffold was covered with a significantly thick lay of HA.

### 3.3. Cell Morphology and Survival

**Figure 4:**
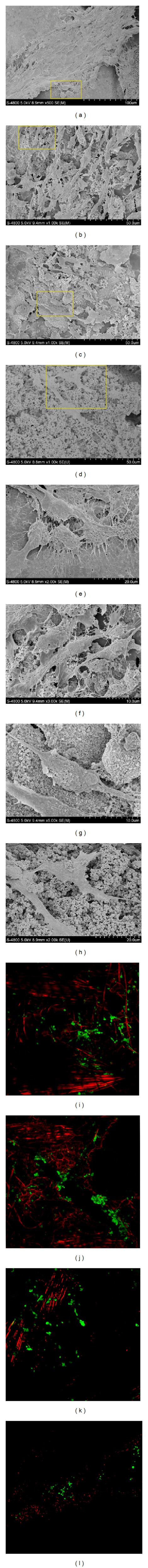
(a)–(d) Morphology and proliferation of cells in four groups observed by SEM after 3-week culture. (e)–(h) Magnified view of rectangle area in (a)–(d). (i)–(l) Cell survival and proliferation observed by confocal laser scanning.

After equivalent amount of osteoblasts was cultured on scaffolds in groups for three weeks, the cell number of 2C-HA (Figure 4(c)) and 3C-HA (Figure 4(d)) groups was much fewer than those of N-HA (Figure 4(a)) and 1C-HA (Figure 4(b)) groups. The cell morphology on scaffolds showed morphology alteration of osteoblasts in N-HA group (Figure 4(e)) compared with that in HA-coated groups (Figures 4(f)–4(h)). Osteoblast cultured in N-HA group had a more spread-out phenotype. The morphology became more longer with increasing HA-coating cycles.

Confocal microscopy observation showed robust cell proliferation and good cell viability (cells with bright green fluorescence dyed by FDA) in N-HA (Figure 4(i)) and 1C-HA groups (Figure 4(j)). The cells were distributed throughout scaffolds and there was no significant cell death (cells with small round bright red fluorescence dyed by PI) in N-HA and 1C-HA groups. But there were a lot of dead cells on scaffolds of 2C-HA (Figure 4(k)) and 3C-HA (Figure 4(l)) groups, especially on the scaffolds of 3C-HA group. The scaffolds possessed autofluorescence, especially red fluorescence. The fluorescence of scaffold was strip or multihole shapes, while that of dead cell was small round shape.

### 3.4. Gene Expression Analyses

**Figure 5:**
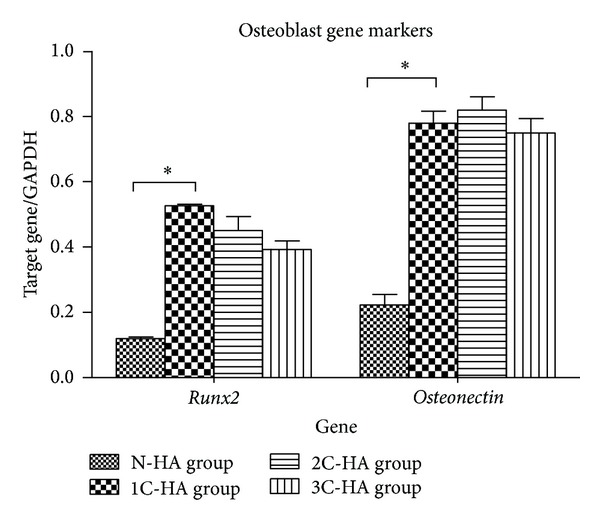
Expression of osteoblast related gene markers after three weeks of culture (*P < 0.05, ANOVA).

Expression of specific genes was analyzed to evaluate the effects of HA modification on the gene expression of osteoblasts. Compared with N-HA group, 1C-HA, 2C-HA and 3C-HA groups showed upregulation of both* runx2* and* osteonectin*. But there was no significant difference among 1C-HA, 2C-HA, and 3C-HA groups (Figure 5).

## 4. Discussion

Alternate soaking technology is a frequently used method to modify the scaffolds with hydroxyapatite. However, dense HA has some obstacles such as non- or poor-osteoinductivity and low rate of biodegradation and porosity [14, 17]. In our pilot study, dense HA-coating was found to inhibit cell proliferation and vitality. In this study, we optimized the thickness of HA-coating. It could benefit both cell proliferation and specific genes expression.

It is well known that the pore size and interconnectivity of scaffolds are highly relevant to proper cell migration and proliferation as well as tissue vascularization and diffusion of nutrients and oxygen, which is necessary for bone formation. Previous studies also demonstrated that pore size between 100 and 350 *μ*m is optimum for bone regeneration [18]. If the pore size is too large, cells may leak out from pores. If pore size is too small, cells may be unable to stretch out. Both too large and too small pore sizes may be not conducive to proliferation and differentiation of cells. Interconnected porosity can maximize bone in growth, lead to osteointegration, and strengthen graft fixation due to larger surface area and more directional in growth of bone. A number of techniques are in practice for the fabrication of porous HA, such as addition of organic porogens [19], polymer foam impregnation [20], dual-phase mixing [21], gel casting [22], and freeze casting [23]. The silk scaffold has a very good microporous structure (Figure 3(e)). Alternate soaking technology with 1 cycle can result in a thin HA layer on scaffold and less destroy the porous structure of scaffold (Figure 3(f)). However, the HA-coating was too thick and the pores were almost filled in 2C-HA and 3C-HA groups. It is not good for cell proliferation and metabolism on scaffolds. Because the interconnectivity of micropores in scaffold was not broken in 1C-HA group, the cell proliferation of 1C-HA group had no significant difference compared with that of N-HA group (Figure 2(a)) according to results of Alamar Blue assay. However, the value of 1C-HA group was significantly higher than that of 2C-HA and 3C-HA groups with much thicker HA-coating (Figure 2(a)). The results of total content of DNA and collagen also showed that cell proliferation of 1C-HA group was obviously higher than those of 2C-HA and 3C-HA groups (Figures 2(b) and 2(c)) after 2 weeks of culture.

Scaffolds modified with HA may not only overcome the poor mechanical properties of HA but also improve the osteogenicity of scaffold. For HA-coating, a variety of methods have been reported: plasma spraying [24], ion beam assisted deposition [25], magnetron sputtering [26], sol-gel processes [27], composite glass coating [28], electrochemical deposition [29–32], and alkaline treatment [33]. Some nature polymers, such as collagen [34, 35], chitosan [36], chitin [37], alginate [38], and silk [39], are employed for combination with HA to apply in bone tissue engineering. In this study, we found that the expression of osteoblasts-related genes in 1C-HA group was dramatically higher than that of N-HA group (Figure 5). This indicated that HA in extracellular matrix could stimulate the osteogenicity of osteoblasts, support the osteoblasts growth, and maintain the properties of mature. However, there was no significant difference in genes expression (Figure 5) of osteoblasts and collagen production (Figure 2(d)) among 1C-HA, 2C-HA, and 3C-HA groups. This indicated that the capability of osteoinduction had no significant difference among groups. It was not correlated with the thickness of HA-coating.

## 5. Conclusion

HA-coating with 1 cycle of alternate soaking can modify the scaffold with appropriate thickness of coating. It could enhance not only the proliferation of osteoblasts but also osteogenicity of scaffolds. The HA-coated silk scaffold might have great potentials in clinical applications.

## Figures and Tables

**Table 1:** Primer sequences for the real-time RT-PCR.

Primer	Sequences
*GAPDH *	F: GAC ATC AAG AAG GTG GTG AAG C
	R: CTT CAC AAA GTG GTC ATT GAG G

*Runx2 *	F: CCT TCC ACT CTC AGT AAG AAG A
	R: TAA GTA AAG GTG GCT GGA TAG T

*Osteonectin (ON) *	F: GAA GTT GAG GAA ACC GAA GA
	R: GGC AGG AGG AGT CGA AG
